# Dominant *RDH12*-retinitis pigmentosa impairs photoreceptor development and implicates cone involvement in retinal organoids

**DOI:** 10.3389/fcell.2025.1511066

**Published:** 2025-04-29

**Authors:** Cécile Méjécase, Ya Zhou, Nicholas Owen, Pablo Soro-Barrio, Riccardo Cheloni, Neelima Nair, Hajrah Sarkar, Lyes Toualbi, Mariya Moosajee

**Affiliations:** ^1^ Development, Ageing and Disease, UCL Institute of Ophthalmology, London, United Kingdom; ^2^ Ocular Genomics and Therapeutics, The Francis Crick Institute, London, United Kingdom; ^3^ Electron Microscopy Science Technology Platform, The Francis Crick Institute, London, United Kingdom; ^4^ Bioinformatics and Biostatistics Science Technology Platform, The Francis Crick Institute, London, United Kingdom; ^5^ Department of Genetics, Moorfields Eye Hospital NHS Foundation Trust, London, United Kingdom

**Keywords:** retinal dehydrogenase 12, autosomal dominant, retinitis pigmentosa, retinal organoids, induced pluripotent stem cells, vitamin A

## Abstract

**Introduction:**

Retinal dehydrogenase 12 (RDH12) is a photoreceptor NADPH-dependent retinal reductase enzyme, converting all-*trans*-retinal to all-*trans*-retinol. Heterozygous variants in *RDH12* cause a rare autosomal dominant (AD) retinitis pigmentosa.

**Methods:**

As no disease models exist, we generated human induced pluripotent stem cell-derived retinal organoids (RO) from an *RDH12*-AD patient (with pathogenic variant c.759del, p.Phe254Leufs*24) alongside a healthy, unaffected control.

**Results:**

*RDH12*-AD RO exhibited correct localization of *RDH12* to the photoreceptor inner segments up to week 44; however, transmission electron microscopy at week 37 showed that photoreceptors were less abundant and shorter in length. Visual cone function, retinol biosynthesis, and the vitamin A pathway were also highly disrupted at week 44.

**Discussion:**

Our study is the first to describe an *RDH12*-AD disease model with pathology at later stages of photoreceptor differentiation, in keeping with the milder disease course seen in humans. It provides insights into the etiology and possible targets for future therapeutic development.

## 1 Introduction

Inherited retinal diseases (IRDs) are a genetically and heterogeneous group of non-progressive and progressive sight loss disorders. Among the progressive group, the severity of the clinical phenotype can vary from a severe early onset Leber congenital amaurosis (LCA) to a mild adult-onset retinitis pigmentosa arising from variants in the same causative gene (Georgiou et al., 2021). *RDH12* is a key example; ∼80 biallelic pathogenic variants account for up to 10% of LCA (Sarkar and Moosajee, 2019), but four rare cases of autosomal dominant (AD) late-onset retinitis pigmentosa (RP) have been reported (Fingert et al., 2008; Muthiah et al., 2022; Sarkar et al., 2020).

*RDH12* encodes the retinal dehydrogenase 12 protein, a NADPH-dependent retinal reductase enzyme (Sarkar and Moosajee, 2019). Localized to the inner segment of photoreceptors (Kurth et al., 2007), RDH12 is involved in the visual cycle. In photoreceptor outer segments, rhodopsin interacts with 11-*cis*-retinal, and its photoactivation transforms it into all-*trans*-retinal. Most all-*trans*-retinal is reduced into all-*trans*-retinol by RDH8 in photoreceptor outer segments; the remaining is processed by RDH12 in the inner segment to protect cells from oxidative stress (Sarkar and Moosajee, 2019). RDH12 has also been reported to metabolize medium-chain aldehyde, especially nonanal (Belyaeva et al., 2005), to resist induced toxicity in HEK293 models (Lee et al., 2008).

While few *in vitro* (cell lines overexpression WT or mutated *RDH12*) and *in vivo* (mutant mouse and zebrafish) models exist for autosomal recessive *RDH12*-retinopathies, no animal model has been created for autosomal dominant *RDH12*-related retinitis pigmentosa. The heterozygous *Rdh12* mouse model does not exhibit any retinal phenotype (Kurth et al., 2007; Maeda et al., 2006). Previous studies report retinal organoids derived from patient cells recapitulate the phenotype observed in patients and are a good model for understanding the pathophysiology associated with autosomal dominant retinal disorders (Buskin et al., 2018; Chirco et al., 2021; Gao et al., 2020; Kruczek et al., 2021; Rodrigues et al., 2022). Hence, in this study, we generated and characterized retinal organoid models differentiated from human induced pluripotent stem cells (HiPSC) derived from a patient with heterozygous pathogenic frameshift c.759del p.(Phe254Leufs*24) in *RDH12*, who was affected with dominant mild, late-onset RP (*RDH12*-AD) (Sarkar et al., 2021; Sarkar et al., 2020).

## 2 Materials and methods

### 2.1 Cell culture

HiPSCs were obtained from an RDH12-AD patient described by Sarkar et al. (2020) and an unrelated age- and gender-matched unaffected control (Méjécase et al., 2020; Sarkar et al., 2021). Three clones for each line were expanded, as previously reported (Méjécase et al., 2020; Sarkar et al., 2021). HiPSCs were differentiated into retinal organoids following a feeder-free xeno-free protocol (Reichman et al., 2017). Optic cups were picked between days 21 and 32 and cultured in 6-well plates with 10 ng/mL of human FGF2 for 7 days (Reichman et al., 2017). Each optic cup was selected and cultured in a 96-well plate, with the previously reported protocol to obtain laminated retinal organoids (Gonzalez-Cordero et al., 2017). Retinoic acid (0.5 µM) was maintained until the latest time point (week 44).

### 2.2 Retinal organoid characterization and electron microscopy analysis

Retinal organoids were cryoembedded at two time points (4× week-18 retinal organoids per line obtained from two differentiations and 5× week-44 retinal organoids per clone obtained from up to four differentiations) for immunostaining as previously reported (Reichman et al., 2014). Immunofluorescence staining was performed on 12-µm thick sections. Samples were incubated in a permeabilizing solution (0.5% Triton X-100 and 0.1% Tween 20 with PBS) for 1 h, in a blocking solution (PBS, 0.2% gelatin, and 0.25% Triton X-100) for 1 h at room temperature, and then in primary antibodies solutions diluted in blocking solution (Supplementary Table S1) overnight at 4°C. After washes in PBS with 0.1% Tween 20 solution, sections were incubated with secondary antibodies (Supplementary Table S1) diluted in blocking solution for 1 h at room temperature. Sections were mounted with ProLong Diamond Antifade Mountant with DAPI (Thermo Fisher Scientific). CRX-positive cells with DAPI co-staining were manually counted in a stack of three slides using Fiji to determine the percentage representation. A t-test was performed with p ≤ 0.05 considered statistically significant.

A TUNEL assay was performed using the ApopTag Fluorescein *In situ* Apoptosis Detection Kit (Millipore) per the manufacturer’s instructions. The entire organoid section (19–28 images per stack) was imaged using an LSM710 confocal microscope at magnification ×40, and a maximum intensity projection Z stack was performed for each image (Lane et al., 2020). TUNEL-positive nuclei were counted manually on Fiji, and a t-test was performed, with p ≤ 0.05 considered statistically significant.

Week 44 retinal organoids (4× week-44 retinal organoids per line) were fixed in 4% paraformaldehyde overnight at 4°C, preserved in an increased sucrose gradient (10%–30%, in PBS1X) overnight for each glucose concentration at 4°C. Retinal organoids were then frozen in isopentane at −50°C. Samples and cryosections were kept at −80°C until use. RNAscope was performed to study *RDH12* (1623951-C2), *RBP4* (469781), *RLBP1* (414221), and *ARR3* (486461-C2) mRNA levels, following the manufacturer’s instructions (RNAscope Multiplex Fluorescent Reagent Kit v2), with a target retrieval step for 5 min and protease III steps for 30 min. The entire organoid section was imaged with a Zeiss Invert880 confocal microscope at magnification ×40, and a maximum intensity projection Z stack was performed for each image. Each dot corresponding to one mRNA molecule was counted with a 3D object counter plugin (Bolte and Cordelières, 2006) with Fiji. DAPI nuclei were counted manually to calculate the volume of dots per cell in each image. A t-test was performed with p ≤ 0.05 considered statistically significant.

Two retinal organoids per line (week 37) were fixed with Karnovsky’s fixative (2.5% glutaraldehyde, 1% PFA, 80 mM cacodylate buffer (pH 7.4), and 20 mM NaOH) for 1 h at room temperature in the dark. After washes in 0.2 M sodium cacodylate solution, samples were postfixed in 1% aqueous osmium tetroxide for 1–2 h at room temperature. Serial dehydration with increasing ethanol concentration (50%, 70%, 90%, and 100%) was performed, followed by a 100% propylene oxide bath. Samples were then embedded in 100% EPON resin. The samples were serially sectioned at the thickness of 70 nm using a UC7 ultramicrotome (Leica Microsystems), and sections were picked up on Formvar-coated 2-mm slot copper grids (Gilder Grids). The sections were post-stained with 2% UA and lead citrate, and sections containing the region of interest were imaged using a 120 kV JEM-1400Flash transmission electron microscope with a sCMOS Flash camera (JOEL, United Kingdom). For each organoid per line, three regions 60 µm apart were studied; photoreceptor segments, which could be small outer segment-like structures or inner segments, were counted and measured from the outer limiting membrane (Supplementary Figure S1). As the two structures can be difficult to distinguish due to the section and the angle of the organoid, they were not separated in our measurements. The measurement and counting were performed blind. As no significant difference was observed between the two researchers’ analyses for three blinded regions (Supplementary Figure S1), the remaining analysis (measurement and counting) was performed by only one researcher. A total of 2,097 and 1,238 segments were counted in the unaffected control and *RDH12*-AD samples. Observations can only be drawn from this as statistical analysis is not appropriate for n = 2 retinal organoids per group.

### 2.3 RNA-seq data analysis

At week 44 (day 303), 12 replicates (two organoids per replicate) from two differentiations of two *RDH12*-AD clones and eight replicates from three differentiations from one unaffected control clone were snap frozen. RNA was isolated and quantified, and cDNA was generated from pooled organoids (Qiagen, United Kingdom). cDNA libraries were sequenced on a HiSeq 2500 with paired-end reads of 150 bp using Illumina Stranded Total RNA with Ribo-Zero Plus (Illumina, United Kingdom). Raw reads were quality- and adapter-trimmed using Cutadapt (version 3.4) (Martin, 2011) before alignment. Reads were mapped, and subsequent gene levels were counted using RSEM 1.3.1 (Li and Dewey, 2011) and STAR 2.7.10a (Dobin et al., 2013) against the human genome GRCh38 using annotation release 95, both from Ensembl. Normalization of raw count data and differential expression analysis was performed with the DESeq2 package (version 1.38.3) (Love et al., 2014) within the R programming environment (version 4.2.2) (R Development Core Team, 2008). The pairwise comparison was performed with the contrast function, from which genes differentially expressed (adjusted p-value less than 0.05) between different conditions were determined. Gene lists were used to look for pathways and molecular functions with over-representation analysis using Reactome (Jassal et al., 2020) and Gene Ontology (GO) (Mi et al., 2019). Genes involved in RDH12 pathways were collected from the PathCards database (https://pathcards.genecards.org/, accessed 29 September 2023).

### 2.4 Statistics and reproducibility

All statistical analyses were performed using R for Statistics (https://www.r-project.org/, v4.4.3).

### 2.5 Data availability

The RNA-seq data generated by this study have been deposited in the NCBI Gene Expression Omnibus under the access code GSE271751, including unprocessed FASTQ files and associated gene count matrices.

## 3 Results

### 3.1 Retinal organoid characterization at different time points

**FIGURE 1:**
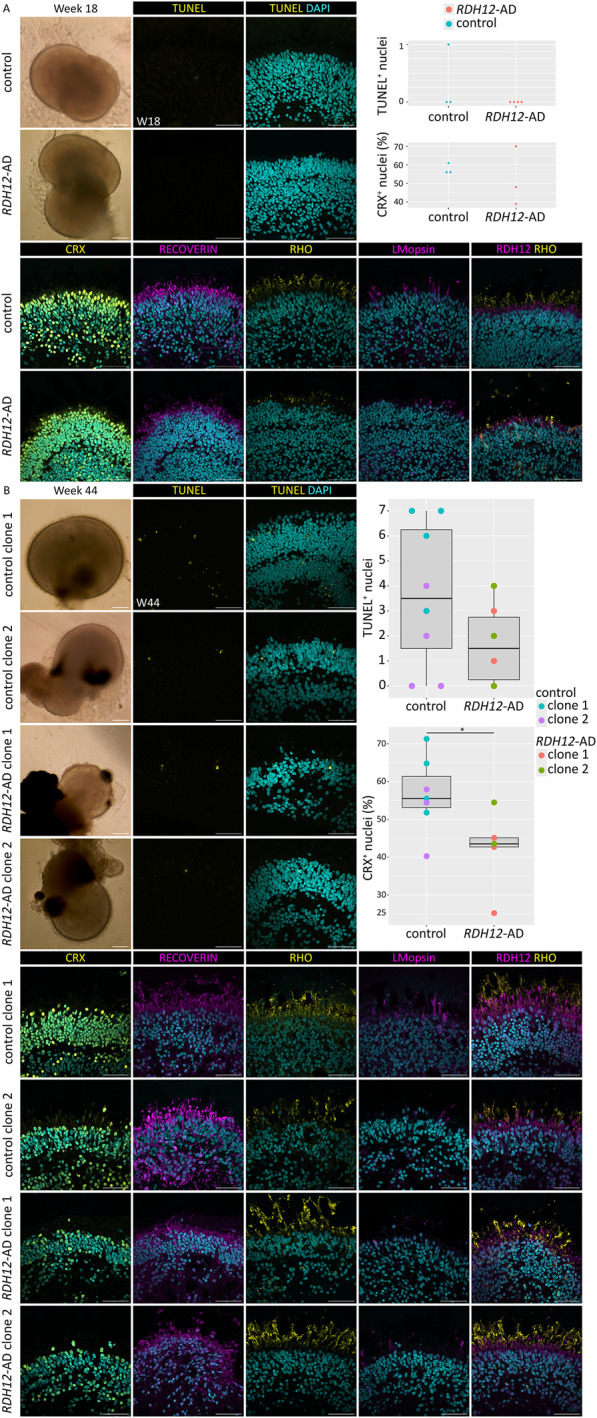
Immunofluorescence studies revealed that *RDH12*-AD retinal organoids developed fewer photoreceptors than unaffected controls. **(A)** *RDH12*-AD and control organoids developed rod and cone photoreceptors at week 18. Three unaffected controls and four *RDH12*-AD retinal organoids were studied. Analysis showed no significant difference in TUNEL^+^ nuclei in *RDH12*-AD organoids compared to control (t-test, p = 0.42) and no significant difference in the percentage of CRX-positive nuclei in *RDH12*-AD organoids compared to control (t-test, p = 0.62). **(B)** At week 44, photoreceptors displayed inner and outer segment-like structures in both control and mutant retinal organoids. Three *RDH12*-AD organoids per clone and five unaffected control organoids at week 44 were studied. Analysis showed no significant difference in TUNEL^+^ nuclei in *RDH12*-AD organoids compared to control (t-test, p = 0.13). *RDH12*-AD retinal organoids had significantly fewer photoreceptors (CRX-positive nuclei) than unaffected controls at week 44 (p = 0.04). CRX, RECOVERIN: photoreceptor precursors; RHO: rod; L/M-opsin: cone. The 200 µm scale bar belongs to brightfield images only; the 50 µm belongs to all the immunostaining images.

HiPSC derived from an *RDH12*-AD patient and an unaffected control were differentiated into retinal organoids and characterized after 18 weeks and 44 weeks of differentiation (Figure 1). At week 18, both unaffected and *RDH12*-AD retinal organoids developed rod and cone photoreceptors, and there was no significant difference in either cell death (p = 0.42) or in the number of photoreceptor cells (CRX-positive cells, p = 0.62, Figure 1A). In both mutant and unaffected control retinal organoids, recoverin was localized in photoreceptor segments, with some recoverin-positive cells detected in the inner nuclear layer, corresponding to bipolar cells. At week 44, there was no significant difference in cell viability (p = 0.13), and both photoreceptor cells grew inner (recoverin-expressing) and outer segment-like structures (with L/M-opsin and rhodopsin expression) (Figure 1B). However, the percentage of photoreceptors (CRX-positive cells) in *RDH12*-AD retinal organoids was significantly decreased compared to the unaffected control at week 44 (p = 0.04, Figure 1B). RDH12 was detected in the photoreceptor inner segments in both the unaffected control and *RDH12*-AD retinal organoids at week 18 and week 44.

### 3.2 Photoreceptors are shorter and less abundant in *RDH12*-AD retinal organoids

**FIGURE 2:**
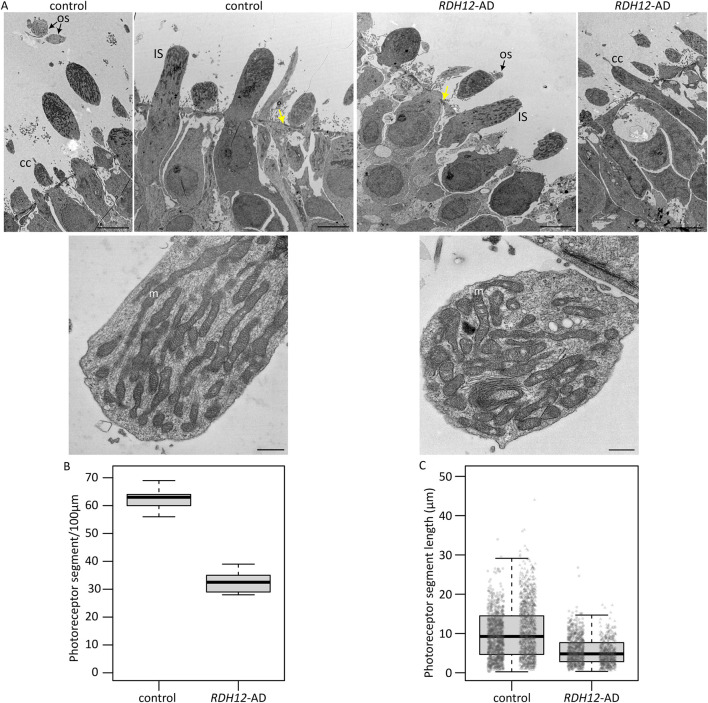
Ultrastructure of *RDH12*-AD mutants and unaffected control retinal organoids at week 37. **(A)** Both unaffected control and *RDH12*-AD retinal organoids developed photoreceptor outer segment (OS) and inner segment (IS)-like structures with mitochondria (m) and connecting cilium (CC). Yellow arrows indicate the outer limiting membrane. Scale bar: 5 µm (top) and 500 nm (bottom). **(B)** *RDH12*-AD retinal organoids had fewer photoreceptors per 100 µm than control organoids. **(C)** Photoreceptor segments were shorter in *RDH12*-AD retinal organoids than in unaffected controls. Data are represented as mean ± SEM. N = 3 regions from 2 organoids per line, from 1 differentiation batch, and a total of n = 2,097 and n = 1,238 photoreceptor segments, from the outer limiting membrane to tip end, were counted and measured in WT and *RDH12*-AD organoids, respectively.

Transmission electron microscopy of week 37 retinal organoids was undertaken to look for intracellular changes (Figure 2). Both unaffected control and *RDH12*-AD organoids developed segments around the organoid edges, with mitochondria and a clear, regular outer limiting membrane (Figure 2A). Small rudimentary outer segment-like structures and connecting cilia were observed in both WT and *RDH12*-AD organoids (Figure 2A). Blinded analyses of three regions per organoid showed unaffected control retinal organoids have an average number of 62 ± 5 photoreceptors per 100 µm whilst *RDH12*-AD have fewer photoreceptors (33 ± 4 photoreceptors per 100 µm) (Figure 2B). The average photoreceptor segment length was 10.09 ± 6.48 µm in unaffected controls (range between 0.25 µm and 44.02 µm) and reduced to 5.51 ± 3.42 µm in *RDH12*-AD retinal organoids (range between 0.36 µm and 26.77 µm) (Figure 2C).

### 3.3 Dominant disease-causing variant in *RDH12* impairs cone functions

**FIGURE 3:**
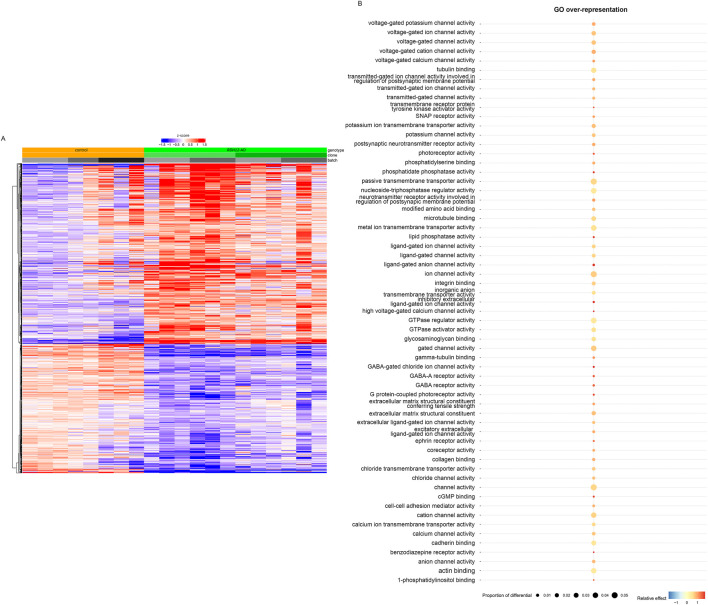
Transcriptomic analyses distinguished *RDH12*-AD mutant from unaffected control retinal organoids, highlighting molecular dysfunction (day 303, week 44). **(A)** Heatmap of differential genes from unaffected control (orange) and *RDH12*-AD (green) mature retinal organoids. Downregulated genes are indicated in blue, and upregulated genes are indicated in red. Both are described with standard deviations around the mean (z-score). **(B)** Gene ontology (GO) analysis identified overrepresented GO molecular function in *RDH12*-AD organoids, including passive transmembrane transporter activity pathways, neurotransmitter receptor activity, nucleoside-triphosphatase regulator activity, photoreceptor activity, lipid phosphatase activity, modified amino acid binding, extracellular matrix structural constituent, glycosaminoglycan binding, transmembrane receptor protein tyrosine kinase activator activity, ephrin receptor activity, cGMP binding, SNAP receptor activity, cell–cell adhesion mediator activity, coreceptor activity, collagen binding, actin binding, cadherin binding, integrin binding, 1-phosphatidylinositol binding, tubulin binding, and microtubule binding. Relative effect indicates under- (below 1) and overrepresented (above 1) pathways, supplemented with a statistically significant (hypergeometric) p-value. The proportion of differential indicates the fraction of the differentially expressed genes that belong to a specific pathway. Bulk RNA sequencing was performed on 12 replicates (two organoids per replicate) from two differentiations of two *RDH12*-AD clones and eight replicates from three differentiations from one control clone.

Bulk RNA sequencing was performed on mature RDH12 and unaffected control retinal organoids at week 44 to identify disease-causing pathways. In mature *RDH12*-AD retinal organoids, 2,446 genes were differentially expressed, with 1,424 upregulated and 1,022 downregulated, compared to the control expression (Figure 3A; Supplementary Table S2; Supplementary Figure S2). Gene ontology (GO) analysis identified GO overrepresentation of passive transmembrane transporter activity pathways (GO:0022803, GO:0005249, GO:0005261, GO:0046873, GO:0015103, GO:0015267, GO:0022836, GO:0005267, GO:0022843, GO:0005244, GO:0022832, GO:0015079, GO:0005216, GO:0022824, GO:0005230, GO:0022834, GO:0005231, GO:0005237, GO:0005245, GO:0005254, GO:0016917, GO:0004890, GO:0022835, GO:0015276, GO:0015085, GO:0005262, GO:0005253, GO:0015108, GO:1904315, GO:0099095, GO:0022851, and GO:0008331), neurotransmitter receptor activity (GO:0098960, GO:0099529, and GO:0008503), nucleoside-triphosphatase regulator activity (GO:0060589, GO:0030695, and GO:0005096), photoreceptor activity (GO:0009881 and GO:0008020), lipid phosphatase activity (GO:0042577 and GO:0008195), modified amino acid binding (GO:0072341 and GO:0001786), extracellular matrix structural constituent (GO:0005201 and GO:0030020), glycosaminoglycan binding (GO:0005539), transmembrane receptor protein tyrosine kinase activator activity (GO:0030297), ephrin receptor activity (GO:0005003), cGMP binding (GO:0030553), SNAP receptor activity (GO:0005484), cell–cell adhesion mediator activity (GO:0098632), coreceptor activity (GO:0015026), collagen binding (GO:0005518), actin binding (GO:0003779), cadherin binding (GO:0045296), integrin binding (GO:0005178), 1-phosphatidylinositol binding (GO:0005545), tubulin binding (GO:0015631 and GO:0043015), and microtubule binding (GO:0008017) in *RDH12*-AD (Figure 3B; Supplementary Table S3).

**FIGURE 4:**
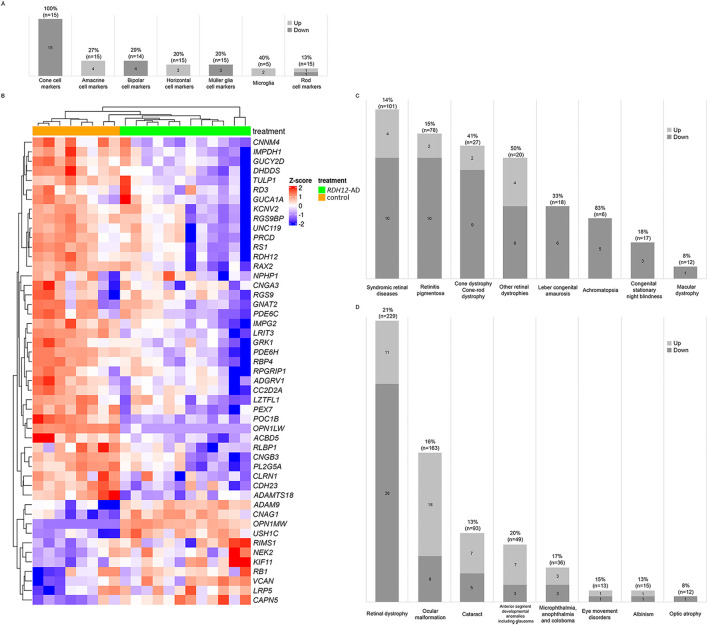
Retinal markers and genes associated with inherited eye diseases were dysregulated in *RDH12*-AD retinal organoids at day 303 (week 44). **(A)** Cone-specific cell markers were downregulated in *RDH12-*AD retinal organoids compared to unaffected controls. **(B, C)** Forty-nine unique genes associated with retinal dystrophies were significantly differentially expressed. **(D)** In total, 79 unique genes associated with inherited eye diseases were significantly dysregulated in *RDH12*-AD retinal organoids. Bulk RNA sequencing was performed on 12 replicates (two organoids per replicate) from two differentiations of two *RDH12*-AD clones and eight replicates from three differentiations from one control clone. Downregulated genes are indicated in blue, and upregulated genes are indicated in red. Both are described with standard deviations around the mean (z-score).

We examined different retinal cell markers (Kim et al., 2023) and found cone-specific markers were downregulated in *RDH12*-AD retinal organoids compared to the unaffected control: *OPN1LW*, *TDRG1*, *GRK7*, *RAB41*, *MYL4*, *PDE6H*, *CNGB3*, *PDE6C*, *GNAT2*, *GUCA1C*, *GNGT2*, *ARR3*, *SLC24A2*, *KCNB2*, and *PEX5L* (Supplementary Table S4; Figure 4A). However, only two stress and/or apoptosis markers were differentially expressed: *MOAP1* (LFC −0.804, padj < 2.109 × 10^−8^) and *AIFM2* (LFC 0.639, padj < 3.276 × 10^−2^) (Deng et al., 2018) (Supplementary Table S4).

We compared the expression of genes previously reported to be associated with inherited eye diseases (Supplementary Table S5; Figure 4). Interestingly, 49 genes associated with retinal dystrophies were significantly differentially expressed in *RDH12*-AD (21%, n = 229), with 38 downregulated and 11 upregulated (Figures 4B–D; Supplementary Table S5). These genes are associated with syndromic retinal disorders (*ACBD5*, *ADAMTS18*, *ADGRV1*, *CAPN5*, *CC2D2A*, *CDH23*, *CLRN1*, *KIF11*, *LZTFL1*, *NPHP1*, *PEX7*, *POC1B*, *USH1C*, and *VCAN*), retinitis pigmentosa (*ADAMTS18*, *CC2D2A*, *CLRN1*, *CNGA1*, *DHDDS*, *IMPDH1*, *IMPG2*, *NEK2*, *PRCD*, *RDH12*, *RLBP1*, and *TULP1*), other retinal dystrophies (*CAPN5*, *LRP5*, *OPN1LW*, *OPN1MW*, *PLA2G5*, *RB1*, *RBP4*, *RGS9*, *RGS9BP*, and *RS1*), Leber congenital amaurosis (*GUCY2D*, *IMPDH1*, *RD3*, *RDH12*, *RPGRIP1*, and *TULP1*), cone dystrophy/cone-rod dystrophy (*ADAM9*, *CNNM4*, *GUCA1A*, *GUCY2D*, *KCNV2*, *PDE6C*, *POC1B*, *RAX2*, *RIMS1*, *RPGRIP1*, and *UNC119*), achromatopsia (*CNGA3*, *CNGB3*, *GNAT2*, *PDE6C*, and *PDE6H*), congenital stationary night blindness (*LRIT3*, *GRK1*, and *RLBP1*), and macular dystrophy (*RAX2*). Differential gene expression was also observed for the different groups of ocular diseases (Figure 4D; Supplementary Table S5): 26 genes are associated with ocular malformations (16%, n = 163), with 18 upregulated and eight downregulated; 12 are associated with cataracts (13%, n = 93), with seven upregulated and five downregulated; 10 are associated with anterior segment developmental anomalies including glaucoma (20%, n = 49), with seven upregulated and three downregulated; six associated with microphthalmia, anophthalmia, and coloboma (17%, n = 36), with three upregulated and three downregulated; two are associated with eye movement disorders (15%, n = 13), with one upregulated and one downregulated; two are associated with albinism (13%, n = 15), with one upregulated and one downregulated; and one gene associated with optic atrophy was downregulated (8%, n = 12).

**FIGURE 5:**
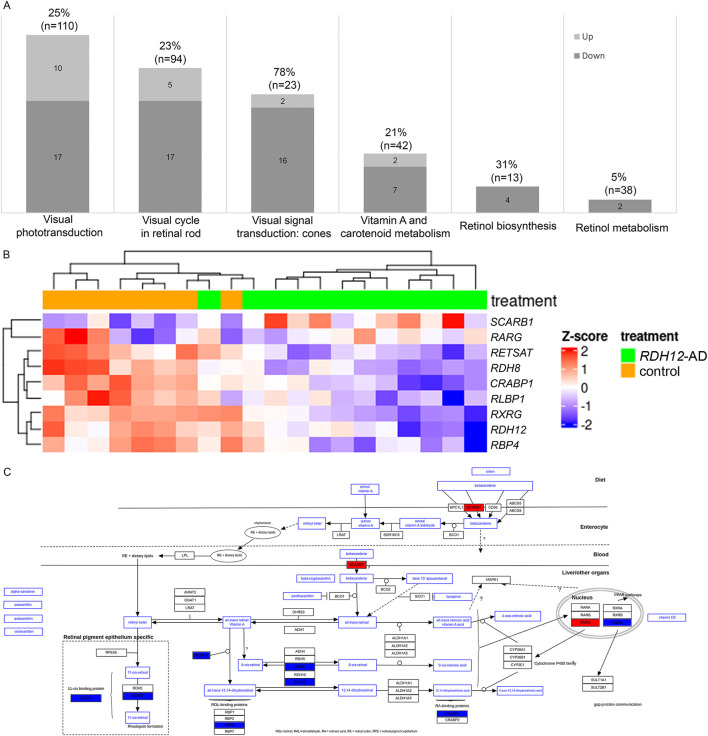
RDH12 pathways were differentially expressed in *RDH12*-AD retinal organoids at day 303 (week 44). **(A)** Forty-five genes involved in RDH12 pathways were significantly differentially expressed. Genes involved in RDH12 pathways were obtained from the PathCards database (https://pathcards.genecards.org/, accessed 29 September 2023). **(B, C)** The vitamin A pathway was affected in *RDH12*-AD retinal organoids at day 303 (W44). Downregulated genes are indicated in blue, and upregulated genes are indicated in red. Both are described with standard deviations around the mean (z-score). Bulk RNA sequencing was performed on 12 replicates (two organoids per replicate) from two differentiations of two *RDH12*-AD clones and eight replicates from three differentiations from one control clone. The scheme was adapted from the WikiPathways website (https://www.wikipathways.org/pathways/WP716.html, accessed on 11 November 2023) (Agrawal et al., 2024; Waagmeester et al., 2009).

*RDH12* expression is downregulated in *RDH12*-AD retinal organoids compared to the unaffected control (LFC −1.158, padj < 4.633 × 10^−3^), and this was supported by RNA *in situ* hybridization (Supplementary Table S6; Supplementary Figure S3). *RDH12* dysregulation may affect several pathways involving RDH12: the visual cycle in rod and cone photoreceptors, vitamin A and carotenoid metabolism, and retinol biosynthesis and metabolism (Sarkar and Moosajee, 2019). Interestingly, 45 unique genes involved in these pathways were dysregulated in *RDH12*-AD retinal organoids compared to the unaffected control (Figure 5A; Supplementary Table S6): of 18 genes involved in visual signal transduction in cones (78%, n = 23), 16 are downregulated, and two are upregulated; of 27 genes involved in visual phototransduction (25%, n = 110), 17 are downregulated, and 10 are upregulated; of 22 genes involved in the visual cycle in retinal rod (23%, n = 94), 17 are downregulated, and five are upregulated; of nine genes involved in vitamin A and carotenoid metabolism (21%, n = 42), seven are downregulated, and two are upregulated; two genes involved in retinol metabolism (5%, n = 38) are downregulated; and four genes involved in retinol biosynthesis are downregulated (31%, n = 13). Cone function appeared to be highly affected in *RDH12*-AD retinal organoids (Supplementary Table S6): *GRK7* (LFC −1.974, padj < 2.061 × 10^−10^), *OPN1MW3* (LFC −1.746, padj < 4.244 × 10^−3^), *PDE6H* (LFC −1.541, padj < 2.014 × 10^−10^), *CNGB3* (LFC −1.514, padj < 4.937 × 10^−5^), *PDE6C* (LFC −1.509, padj < 1.042 × 10^−12^), *GNAT2* (LFC −1.499, padj < 8.770 × 10^−9^), *GNGT2* (LFC −1.447, padj < 1.042 × 10^−5^), *ARR3* (LFC −1.438, padj < 1.528 × 10^−6^), *SLC24A2* (LFC −1.403, padj < 1.079 × 10^−3^), *GNB3* (LFC −1.202, padj < 6.145 × 10^−4^), *RDH12* (LFC −1.158, padj < 4.633 × 10^−3^), *GRK1* (LFC −1.132, padj < 6.192 × 10^−5^), *RGS9BP* (LFC −0.886, padj < 2.274 × 10^−3^), *CNGA3* (LFC −0.824, padj < 2.127 × 10^−2^), *GUCY2D* (LFC −0.788, padj < 2.388 × 10^−3^), *RGS9* (LFC −0.589, padj < 3.590 × 10^−2^); and *OPN1MW* (LFC 10.42, padj < 3.188 × 10^−39^), and *OPN1MW2* (LFC 10.658, padj < 2.172 × 10^−25^). RNA *in situ* hybridization confirmed that *RLBP1*, a marker of the vitamin A pathway, was downregulated in the *RDH12*-AD retinal organoids compared to unaffected controls (p = 0.009, Supplementary Figure S3B). A trend toward reduction was seen with *RBP4*, which is involved in retinol biosynthesis (p = 0.121, Supplementary Figure S3C), and with *ARR3*, involved in cone phototransduction (p = 0.275, Supplementary Figure S3). The vitamin A pathway was downregulated in *RDH12*-AD retinal organoids (Figures 5B,C).

## 4 Discussion

This study describes the first retinal organoid model for autosomal dominant *RDH12*-related disease, derived from a patient with a heterozygous frameshift variant c.759del p.(Phe254Leufs*24). This powerful tool unravels disease mechanisms associated with a milder and late-onset retinitis pigmentosa, providing further insights into RDH12 function in the retina.

Autosomal dominant *RDH12* retinopathy is considered a milder form of IRD with relatively late onset in adulthood. It is characterized by nyctalopia and progressive visual field loss with associated bone spicules in the peripheral retina and retinal vessel attenuation. The central vision is affected much later in the disease, although the number of peripheral cone photoreceptors is reduced, and their outer segment is abnormal (Fingert et al., 2008; Muthiah et al., 2022; Sarkar et al., 2020). *RDH12*-AD retinal organoids model the mild phenotype seen in patients; they display a similar development process to unaffected control organoids with production of the outer nuclear layer with mature photoreceptors, albeit with shorter photoreceptor segments and reduced photoreceptor cell numbers. Variants in *LRIT3*, *GRK1*, and *RLBP1* were previously reported to be associated with autosomal recessive or X-linked congenital stationary night blindness (Zeitz et al., 2015). Their effect was predicted to produce misfolded non-functional protein or decreased expression (Damodar et al., 2024; Poulter et al., 2021; Zeitz et al., 2013). Interestingly, these genes were downregulated in the *RDH12*-AD retinal organoids. Moreover, cone-specific markers associated with predominantly cone diseases (achromatopsia, cone/cone-rod dystrophy, and macular dystrophy) were downregulated, suggesting cones are preferentially affected in *RDH12*-AD models. In addition, 56 genes associated with ocular size, corneal defects, and cataracts were dysregulated, with 19 downregulated, suggesting a link to the hypermetropic refractive error commonly seen in RDH12 patients and associated complications seen with RP (Aleman et al., 2018). Further transcriptomics studies on IRD gene expression in different RP models may uncover common disease pathways.

Mature retinal organoids resemble more peripheral retina than macula (Cowan et al., 2020), with a well-delineated outer limiting membrane and photoreceptor segments exhibiting inner and outer segment characteristics, mitochondria, and discs (Zhong et al., 2014). Ultra-structural and immunostaining analysis of *RDH12*-AD retinal organoids revealed shorter photoreceptor segments and reduced cellular numbers than unaffected controls, consistent with shorter cone inner segments and a decreased cone density seen through adaptive optic scanning light ophthalmoscopy (AOSLO) in affected patients (Fingert et al., 2008; Muthiah et al., 2022; Sarkar et al., 2020). The number of photoreceptors at week 18 in *RDH12-*AD retinal organoids is similar to unaffected control organoids, showing cell death may have occurred between weeks 18 and 38, thus excluding any developmental defect in the *RHD12-*AD model. While cone defects are observed later in the disease, they cannot be excluded at earlier stages when the patient is potentially pre-symptomatic. Moreover, transmission electronic microscopy of retinal organoids cannot clarify which type of photoreceptors, rods or cones, are impacted. Shortened segments are an early sign of photoreceptor dysfunction and degeneration in RP patients (Milam et al., 1998) and in several mouse models (*rds* and *rd*; Travis, 1998). There was no change in the ultrastructure of cellular organelles, and immunostaining at various time points failed to detect a significant increase in cell death in *RDH12*-AD organoids. Moreover, in week 44 *RDH12*-AD retinal organoids, two pro-apoptotic genes, *MOAP1* and *AIFM2*, were downregulated and upregulated, respectively (Su et al., 2022; Wu et al., 2002). The equilibrium of these genes might be altered in some cells, leading to their death and thus reducing the number of photoreceptors. Photoreceptor cell death may have occurred between weeks 18–38, time points not examined in this study; hence, further ultrastructure analyses at different time points during photoreceptor maturation would be needed to assess for longitudinal changes.

*RDH12*-AD retinal organoids are derived from patient cells, heterozygous for c.759del p.(Phe254Leufs*24) in *RDH12* (Sarkar et al., 2021; Sarkar et al., 2020). This frameshift variant creates a premature termination codon (PTC) located 19 nucleotides upstream of the last exon–exon junction in exon 8, predicted to be resistant to nonsense-mediated decay (NMD) (Kurosaki et al., 2019). Transcriptomic analysis reveals that *RDH12* expression decreased by 45% in *RDH12*-AD organoids compared to the unaffected control. The presence of mutated *RDH12* mRNA in mutant retinal organoids suggests a partial degradation. The mutated *RDH12* mRNA, which is insensitive to NMD, produces a 40-amino-acid shorter protein, with a 17-amino-acid sequence shared with the two other variants previously reported in autosomal dominant *RDH12*-retinal diseases (Fingert et al., 2008; Muthiah et al., 2022). The WT *RDH12* allele localized to the photoreceptor inner segments (Kurth et al., 2007), including in *RDH12*-AD retinal organoids, was noted at week 18 and week 44. RDH12 was suggested to function as a dimer based on the ortholog photoreceptor retinal dehydrogenase in *Drosophila melanogaster* (Sarkar and Moosajee, 2019). The alternative C-terminus may function as a dominant-negative protein, sequestering WT protein to reduce its activity.

*RDH12*-AD-related RP is characterized by a late-onset milder phenotype as observed in patients affected with *PDE6B*-RP (Kim et al., 2020; Sarkar et al., 2020; Sarkar and Moosajee, 2019). *RDH12* and *PDE6B* are both involved in the vision signal transduction pathway. *PDE6B* expression is unchanged in mature *RDH12*-AD organoids where *RDH12* is downregulated, while *RDH12* is upregulated in *PDE6B* mutant organoids with a missense variant leading to gene upregulation (Gao et al., 2020). Phototransduction in both *PDE6B* and *RDH12*-AD retinal organoid models reveal downregulation of *ARR3*, *CNGA3*, *GNAT2*, *OPN1LW*, *PDE6C*, *PDE6H*, *RGS9BP*, and *RPGRIP1* (Gao et al., 2020). Further analysis comparing transcriptomic signatures in both RP models to investigate common pathways could be interesting. Transcriptomic analyses at week-44 *RDH12*-AD retinal organoids reveal 79 inherited eye disease-associated genes are differentially expressed, with 47 genes downregulated, including 49 associated with IRDs. None of these genes have previously been modeled with patient-derived iPSC to study their effect on the retina, but transcriptomic analysis of such retinal organoids may highlight common pathways that could be targeted for more agnostic therapeutic approaches. Of note, *RDH12* expression is restricted to the retina, especially cone and rod photoreceptors (Cowan et al., 2020), but the visual cycle occurs in the retina and the RPE (Kiser et al., 2014). Amongst RPE proteins involved in the visual cycle, LRAT converts all-*trans*-retinol into all-*trans*-retinaldehyde, which is transformed into 11-*cis*-retinol by RPE65. RDH5 catalyzes 11-*cis*-retinal biosynthesis from 11-*cis*-retinol before retinoid transport back into photoreceptors to start a new phototransduction cycle. Mutations in *LRAT* or *RPE65* are associated with Leber congenital amaurosis, and variants in *RDH5* lead to *fundus albipunctatus*. Presently, differentiation and culture protocols do not effectively co-culture retinal organoids with RPE (Fathi et al., 2021), limiting the complete identification of affected pathways in AD *RDH12-*RP.

In summary, we have developed the first *RDH12-*AD retinal organoid model and highlighted photoreceptor dysfunction and vitamin A pathway defects. The genetic background is highly important in determining disease pathogenicity and severity; hence, isogenic controls would help validate these results (Riordan and Nadeau, 2017). Only four patients have been reported in the literature with AD *RDH12-*RP (Fingert et al., 2008; Muthiah et al., 2022; Sarkar et al., 2020); one unrelated family sharing the same variant studied here (Sarkar et al., 2020), and two other unrelated families, who were heterozygous for the c.776del p.(Glu260Argfs*18) or c.763del p.(Val255Serfs*23) variants in *RDH12*, respectively (Fingert et al., 2008; Muthiah et al., 2022). Retinal organoid models from each unrelated family or the introduction of the pathogenic variant on the same WT background using CRISPR-Cas9 gene editing would enable further characterization, electron microscopy, and transcriptomic analyses to advance our knowledge of the disease mechanisms associated with *RDH12-*RP.

## Data Availability

The RNA-seq data generated by this study have been deposited in the NCBI Gene Expression Omnibus (https://www.ncbi.nlm.nih.gov/geo/) under the access code GSE271751, including unprocessed FASTQ files and associated gene count matrices.
