# Supplementary figures and images for: Dominant RDH12-retinitis pigmentosa impairs photoreceptor development and implicates cone involvement in retinal organoids
Source: Front Cell Dev Biol. 2025 Apr 29;13:1511066. doi: 10.3389/fcell.2025.1511066 (PMC12069300; doi:10.3389/fcell.2025.1511066)

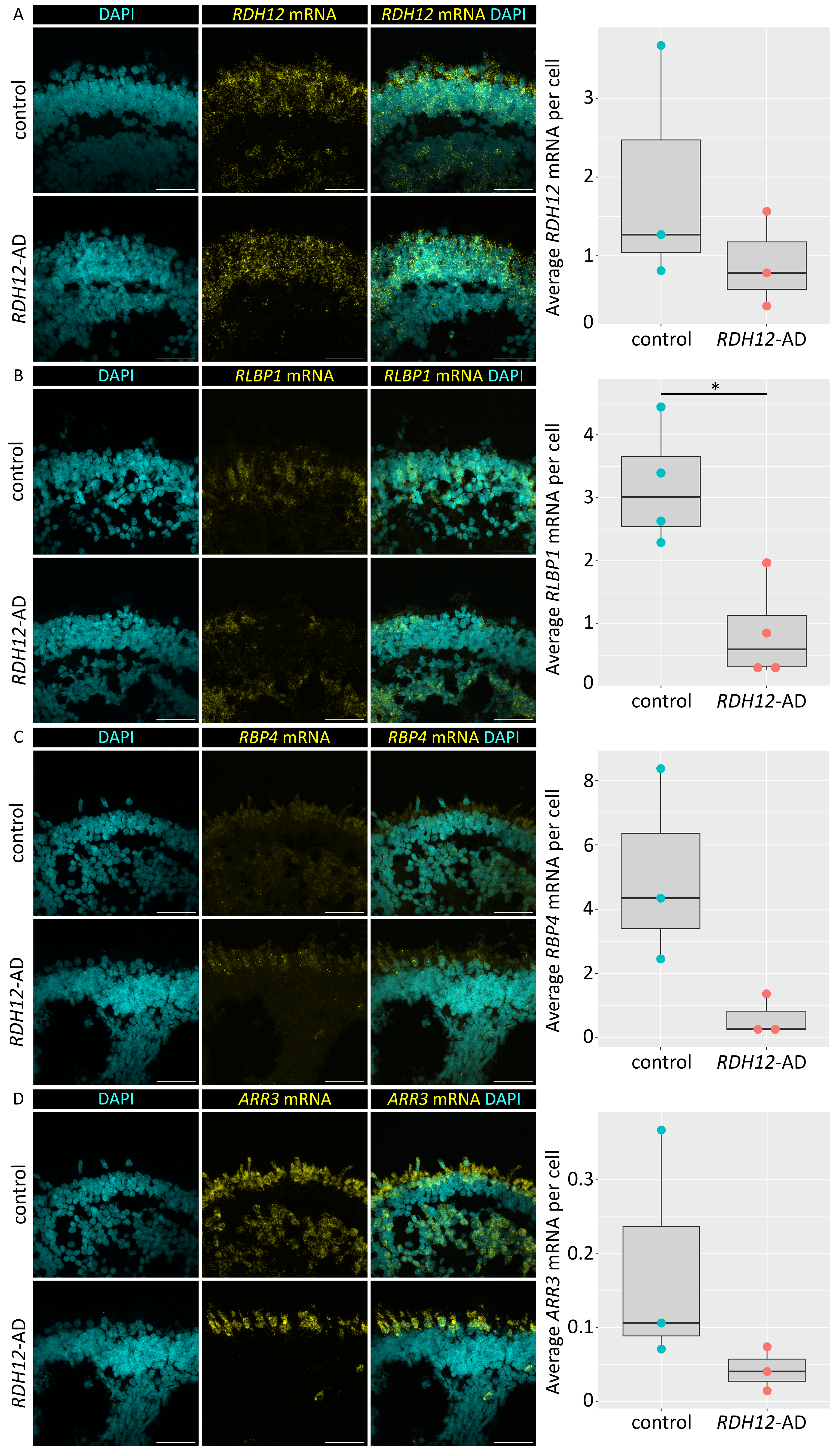

Supplement: Supplementary file 3 [file Image3.tif]

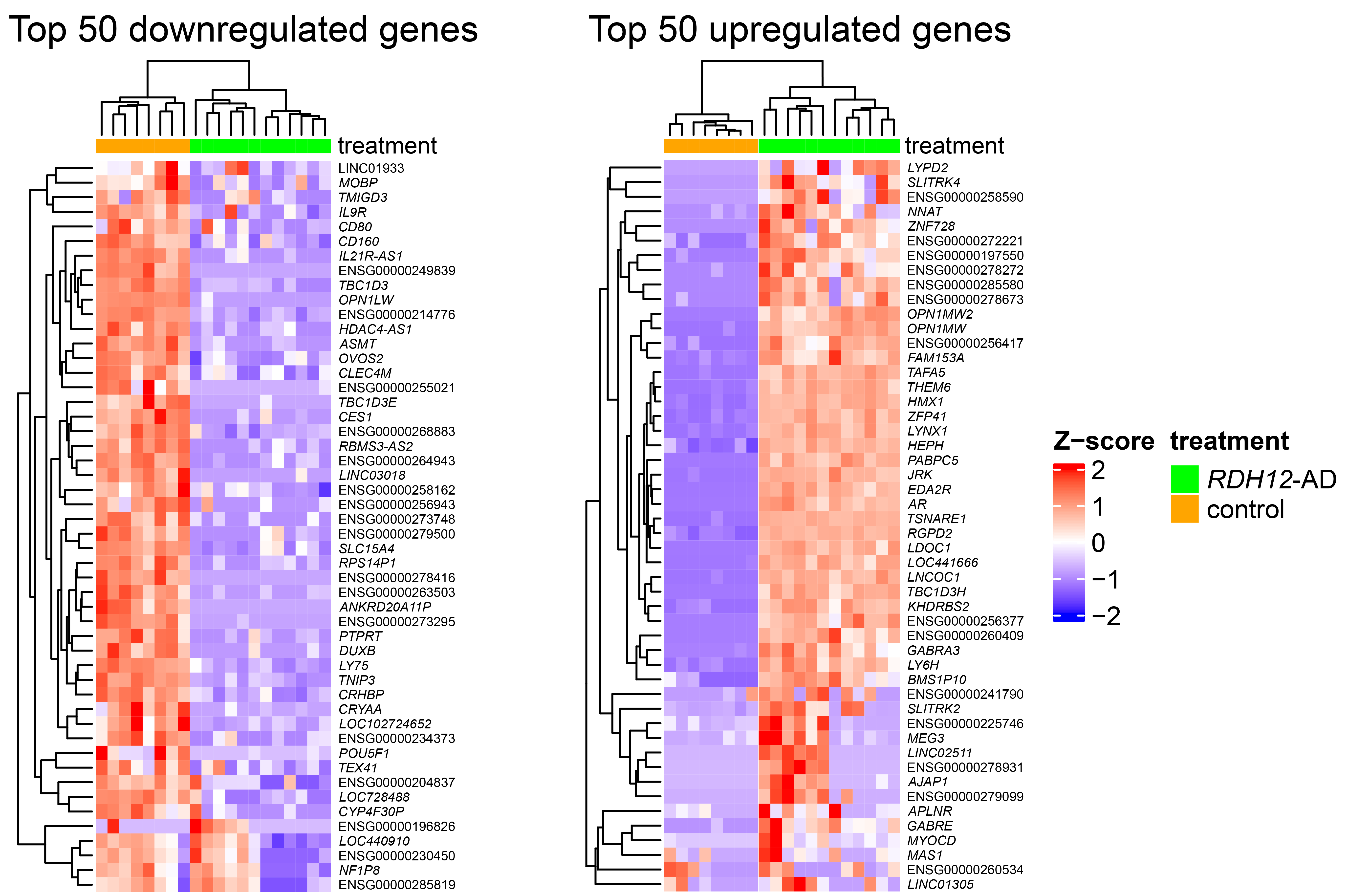

Supplement: Supplementary file 4 [file Image2.tif]

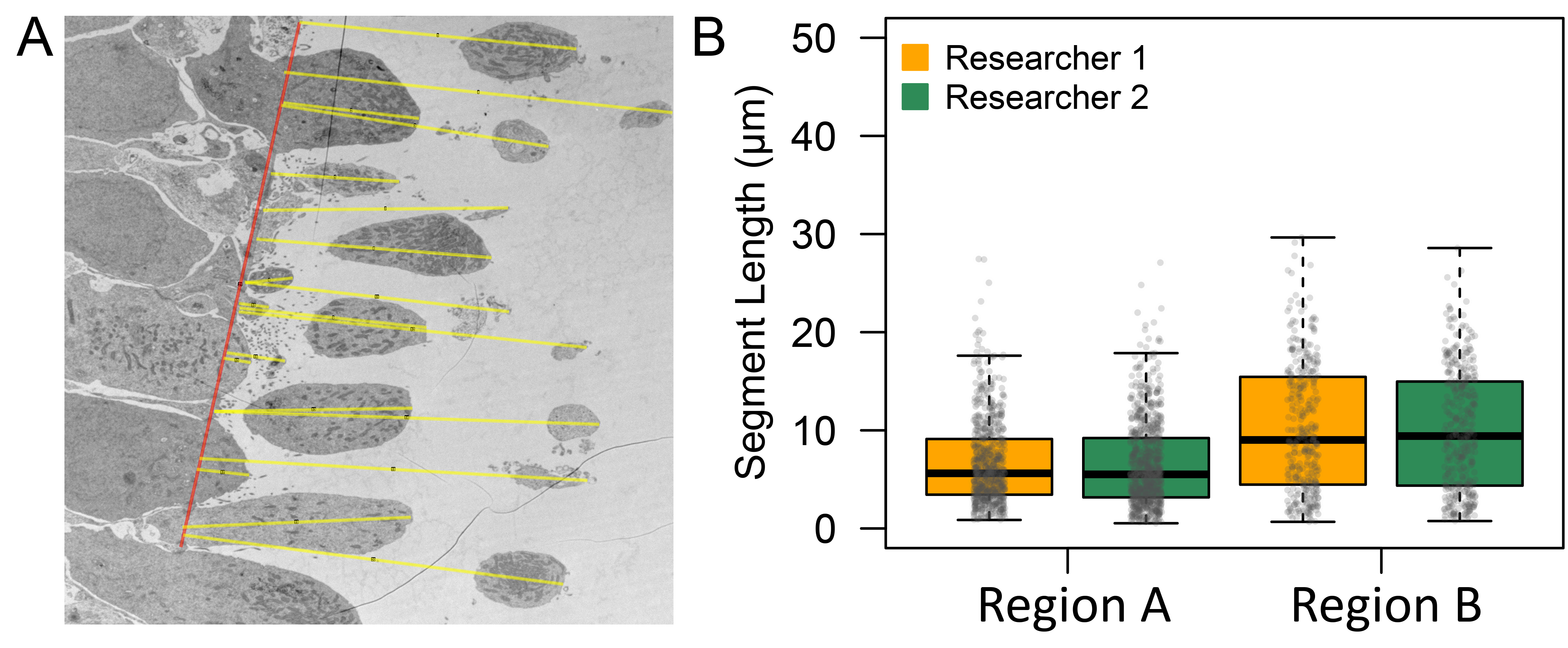

Supplement: Supplementary file 5 [file Image1.tif]
