# Supplementary material for: Dominant RDH12-retinitis pigmentosa impairs photoreceptor development and implicates cone involvement in retinal organoids
Source: Front Cell Dev Biol. 2025 Apr 29;13:1511066. doi: 10.3389/fcell.2025.1511066 (PMC12069300; doi:10.3389/fcell.2025.1511066)
Supplement: Supplementary file 10 [file DataSheet1.docx]

Supplementary Material

**Supplementary Table S1:** Primary and secondary antibodies.

**Supplementary Table S2**: More than 2,000 genes were differentially expressed in mature *RDH12-AD* retinal organoids compared to unaffected controls. * Significantly differentially expressed. Gene identifier was described with its ENSEMBL number (id column), gene name (symbol column) and its NCBI number (entrez column).

**Supplementary Table S3**: Gene ontology (GO) over-represented in *RDH12*-AD retinal organoids.

**Supplementary Table S4:** Retinal cell markers and stress-apoptosis genes were differentially expressed in mature *RDH12*-AD retinal organoids. * means significantly differentially expressed.

**Supplementary Table S5**: Seventy-nine genes associated with inherited eye diseases were significantly differentially expressed in mature *RDH12*-AD retinal organoids. Genes highlight in grey were reported in two or more PanelApp subgroups. * means significantly differentially expressed.

**Supplementary Table S6:** Forty-five genes involved in RDH12 pathways were differentially expressed in mature *RDH12*-AD retinal organoids. Genes highlight in grey were reported in two or more pathways. * means significantly differentially expressed.


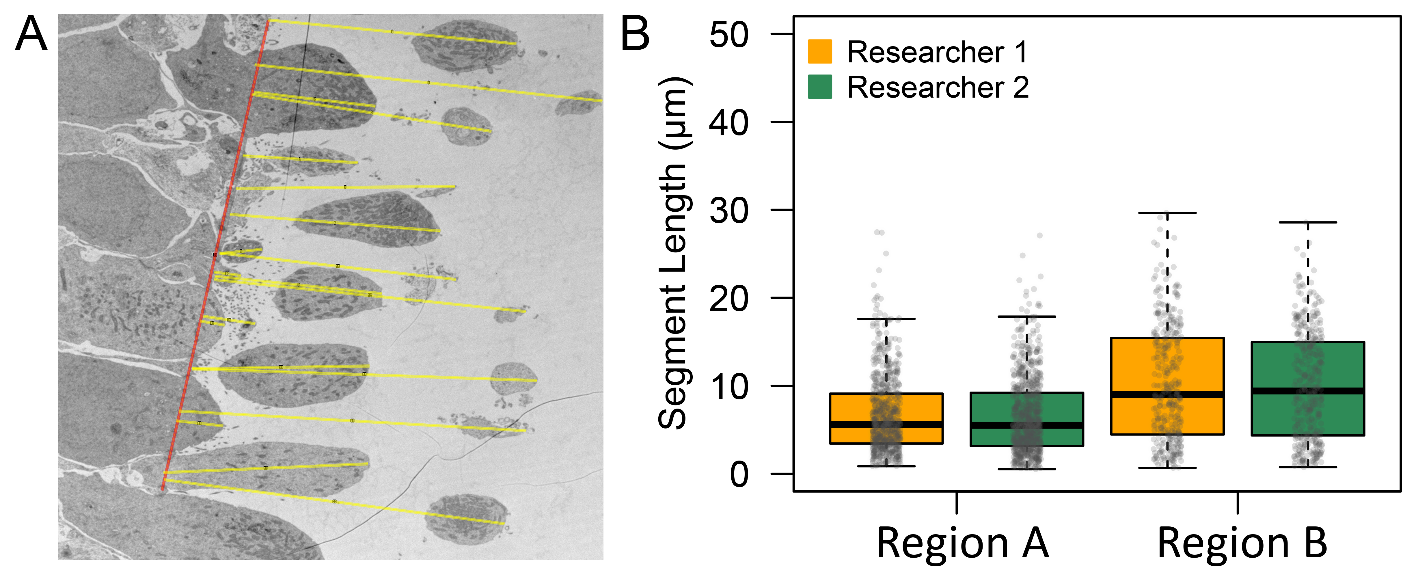


**Supplementary Figure S1: Blind analysis of photoreceptor length and counts analyses was similar between researchers. (A)** Example of photoreceptor count and measurement in transmission electron microscopy images. Red line measured aera where photoreceptors were counted and measured. Each yellow line was counted to determine number of photoreceptors; and measured to determine length between outer limiting membrane and tip end of photoreceptor segment, that could be small outer segment like structures or inner segment length. **(B)** Boxplots reporting changes in segment length across different researcher. Differences were tested with linear mixed model, including segment length as depending variable, researcher as fixed effect and condition, organoid number and region as random effects. Analysis showed no significant difference in segment length by researcher (χ2=0.7, p=0.34).


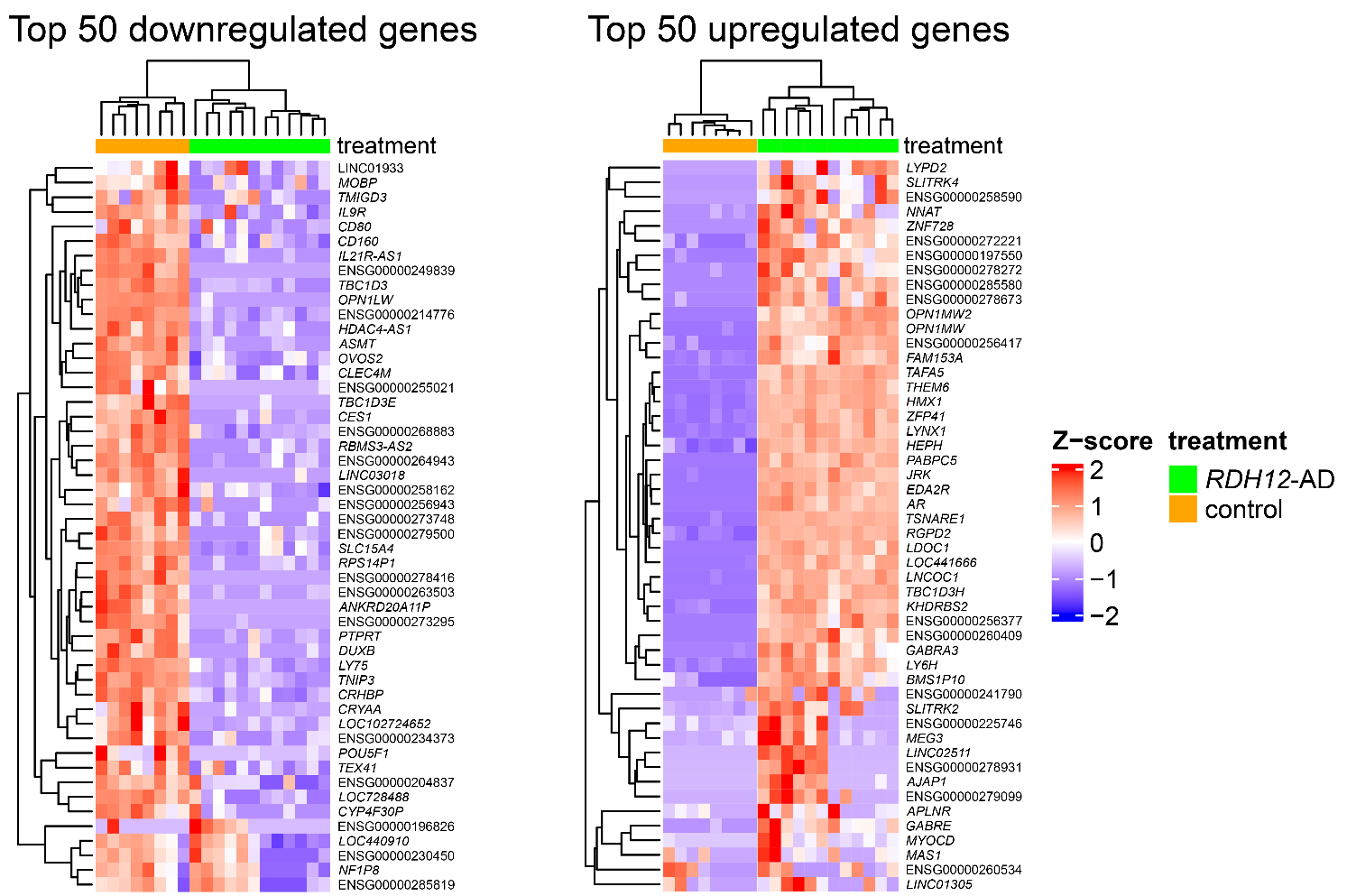


**Supplementary Figure S2: Heatmaps of top 50 downregulated and upregulated genes in *RDH12*-AD mature retinal organoids (*RDH12-*AD) compared to unaffected control (control).** Bulk RNA sequencing was performed on 12 replicates (two organoids per replicate) from two differentiations of two *RDH12*-AD clones and 8 replicates from three differentiations from one control clone. Downregulated genes are indicated in blue and upregulated genes in red, described with standard deviations around the mean (Z-score).


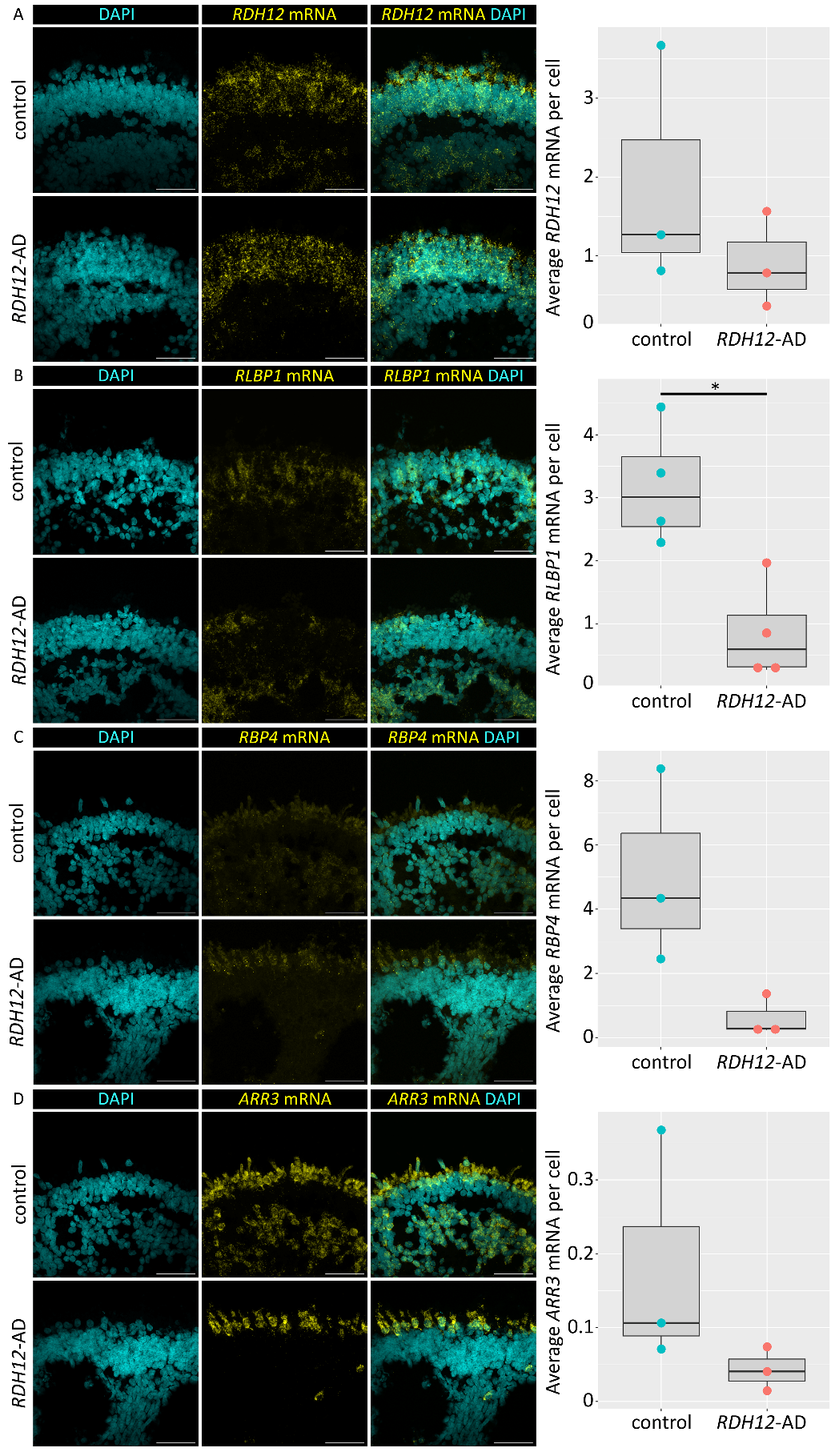


**Supplementary Figure S3: RNA *in situ* hybridization supported bulk RNA sequencing results. (A)***RDH12* mRNA were localised to the outer nuclear layer in both unaffected control and mutant week-44 retinal organoids. *RDH12-*AD retinal organoids showed a trend towards reduction of *RDH12*mRNA expression compared to unaffected controls (t-test, p=0.376). **(B)** *RLBP1*mRNA was localised to a few cells in the outer nuclear layer and mostly in the inner nuclear layer, corresponding to Müller glia cells, in both retinal organoid models. *RLBP1*mRNA levels were significantly decreased in *RDH12-*AD compared to unaffected controls (t-test, * p=0.009). **(C)** *RBP4* mRNA was expressed in photoreceptor inner segments in both retinal organoids models. *RDH12-*AD retinal organoids showed a trend towards reduction of reduced*RBP4*mRNA level compared to unaffected controls (t-test, p=0.121). **(D)** *ARR3* mRNA were localised in cone photoreceptor segments in both retinal organoids models. *RDH12-*AD retinal organoids showed a trend towards reduction of*ARR3*mRNA levels compared to unaffected controls (t-test, p=0.275). Average mRNA expression was determined according to volume of staining per cells. RNA *in situ* hybridization was performed on 4 retinal organoids per line. Scale bar: 50µm.
